# Current News

**Published:** 1901-02

**Authors:** 


### Current News.

### NEW YORK ODONTOLOGICAL SOCIETY.

At the annual meeting of the above Society the following officers were elected for the year 1901: President, Dr. W. W. Walker; Vice-President, Dr. J. F. P. Hodson; Recording Secretary, Dr. John I. Hart; Treasurer, Dr. F. C. Walker; Curator, Dr. J. Adams Bishop; Editor, Dr. J. W. Turner; Corresponding Secretary, Dr. W. D. Tracy.

### RHODE ISLAND DENTAL SOCIETY—RESOLUTION.

*Resolved,* That the Rhode Island Dental Society heartily endorse the work that has been done by Dr. J. N. Crouse as Chairman of the Dental Protective Association, and that we earnestly urge him to continue that good work, which we realize has been accomplished at a personal sacrifice of time and money.

